# Supplementary material for: Mental health inequalities, challenges and support needs during COVID-19: a qualitative study of 14-to-25-year-olds in London
Source: BMJ Open. 2024 Apr 2;14(4):e080161. doi: 10.1136/bmjopen-2023-080161 (PMC10989111; doi:10.1136/bmjopen-2023-080161)
Supplement: Supplementary data [file bmjopen-2023-080161supp001.pdf]

Appendices

Appendix A: Participant Demographics.

	Gender	Age*	Ethnicity
1	F	18-21	Black or Black – African
2	Undisclosed	22-25	White – English / Welsh / Scottish / Northern Irish / Irish
3	M	18-21	Asian – Bangladeshi
4	F	18-21	Mixed – White and Asian
5	F	14-17	Black or Black – African
6	F	14-17	Asian – Indian
7	F	14-17	Any other White background
8	M	18-21	Any other Ethnic Group
9	F	22-25	White – English / Welsh / Scottish / Northern Irish / Irish
10	F	22-25	Any other White background
11	F	18-21	White – English / Welsh / Scottish / Northern Irish / Irish
12	F	18-21	White – English / Welsh / Scottish / Northern Irish / Irish
13	F	14-17	White – English / Welsh / Scottish / Northern Irish / Irish
14	M	14-17	Black or Black – African
15	F	18-21	White – English / Welsh / Scottish / Northern Irish / Irish

16	Undisclosed	18-21	White – English / Welsh / Scottish / Northern Irish / Irish
17	F	18-21	White – English / Welsh / Scottish / Northern Irish / Irish
18	M	18-21	Asian – Bangladeshi
19	M	22-25	White – English / Welsh / Scottish / Northern Irish / Irish
20	M	18-21	White – English / Welsh / Scottish / Northern Irish / Irish

* To maintain anonymity of participants, ages have been presented in the form of age ranges.

Appendix B: Final interview schedule.

Introductions - Researchers will introduce themselves, along with Islington Council team and social worker and any student researchers present. Thank young people for taking part and joining the 1-on-1 interview/focus group. Build rapport. Walk through participant information sheet with young people, read verbatim each item on the consent form to ensure participants understand. Take questions.

Lay groundwork for why we're conducting this project, project aims, what we hope to learn and project outputs.

Express ethical considerations (right to withdraw at any time, don't have to share information they don't want to, don't have to answer all questions, confidentiality, trust, safe space to share, no right or wrong answers). Again, emphasise that researchers are here to learn from young people, in order to help other young people in the future as we navigate out of the COVID pandemic. Remind young people the session will be split into 2 parts and last between 20-30 minutes.

QUESTIONS

Lee J, Wong KK-Y. *BMJ Open* 2024; 14:e080161. doi: 10.1136/bmjopen-2023-080161

Part 1 – barriers to health access, impact of covid on health and changes

1. Thinking over this past year, how has it been for you?
    a. Prod: What are some things you are grateful for, or challenges that you faced?
2. Did you learn anything about yourself or others around you? How were you close relationships?
3. Thinking about this past year, has the pandemic affected the way you trust in others? Institutions, governments, community, people?
4. What were some highlights?
5. How has covid impacted your health – mentally and physically?
6. Thinking about pre-pandemic and now, what were some things that has changed for the better? Or for the worse?
7. Thinking about pre-pandemic and now, how has your health changed for the better? Or for the worse?
8. Thinking about some of your challenges, what are some examples of your struggles/challenges?
9. What did you enjoy or least enjoy during the pandemic?

Part 2 – Support that young people would like or need

1. What support did you receive during the pandemic, if any?
2. How was the support that you received, if any?
    a. How did you come across this support?
    b. What were the barriers to the support? If any?
    c. If no barriers, what made it accessible?
3. If you could get more support during the pandemic – and now – what might you want/need?
4. Thinking specifically about your health (physical/mental), what do you wish you had known? What support would you want?
5. What do you think are the key areas that you need most support on? Where might you go to get support?

6. Was there anything else I didn't ask that you would like to share?

Debrief and thank young people for their time. Inform next steps in project on co-creating workshops together.

Appendix C: NVivo coding framework

Three 'parent' codes were generated from the interviews and focus groups: 'Covid-related', 'general', and 'support needs'. The manuscript focused on 'Covid-related' and 'support needs' (*), with eight themes being generated from their sub-codes. Sub-codes from 'general' were summarised in Appendix E and not discussed in the manuscript.

1. Covid-related*
    a. Covid access to nature
    b. Covid affecting social attitudes and personality
    c. Covid attitudes
    d. Covid caring responsibilities
    e. Covid coping mechanisms
    f. Covid difficulties with school, especially online learning
    g. Covid dissatisfaction or distrust towards government or companies
    h. Covid grief and bereavement loss
    i. Covid habits and routines (including difficulties)
    j. Covid impact on family
    k. Covid impact on friendships
    l. Covid impact on mental health
    m. Covid impact on physical health
    n. Covid leading to loss of opportunity

o. Duality of Covid – also positive impact and experience

2. General

a. Achievements

b. Careers and next steps

c. Desire to empower others

d. Desire to open up and be heard

e. General difficulties at school

f. General mental health difficulties

g. General physical health difficulties

h. General relationship with family

i. General social difficulties

j. Inequality and deprivation

k. Religious customs

l. Role of technology (especially in friendships)

m. Social mistrust

n. Self confidence

3. Support needs*

a. Financial support

b. Support for academics and academic-related decisions

c. Support for forming routines and habits

d. Support for less advantaged and vulnerable groups

e. Support for mental health

f. Support for physical health

g. Support for religious customs

h. Support for self-image and confidence

i. Support from family

j. Support from friends and peers

k. Support in developing skills, especially new and creative skills

Appendix D: Sub-themes with supporting quotes of participants' Covid-19 difficulties and support needs (in addition to quotes in the manuscript).

Table D1. Sub-themes with supporting quotes on participants' Covid-19 difficulties

**Sub-theme**	**Illustrative quotes**
Health (mental health and physical health)	"I think the pandemic has made me anxious about… what other people think about me."<br><br>"self-image, like the way I see myself… it's really just deteriorated because of the pandemic"<br><br>"There was nowhere you could go, you couldn't go for a walk either, to clear your head…"<br><br>"Covid wasn't great for like anyone's mental health… because you just indoors the whole time."<br><br>"I don't feel like I have been the same since Covid... I feel so fatigued. I don't sleep as well anymore."
Relationships	Friendships -<br>"during the pandemic I had arguments with a lot of people... I distanced from everybody"<br>"It was definitely due to the pandemic that I got closer to a lot more people... If it wasn't for the pandemic, I don't think I would have as many friends as I have now."

Lee J, Wong KK-Y. *BMJ Open* 2024; 14:e080161. doi: 10.1136/bmjopen-2023-080161

	"there were group chats and I wasn't involved in it... [they] perceive[d] that I just didn't want to form relationships anymore"<br><br>Family -<br>"Another enjoyable thing was probably getting with the family... really having a bonding moment"<br>"I wasn't very close to my family and I distanced from them"
Habits and routines	"there was definitely more… overeating"<br><br>"I wasn't going to school and I didn't have like, a proper routine."<br><br>"I was on my phone all the time like I had no like everyday distractions... heavy use of social media during that time."<br><br>"I typically have a hard time focusing on anything in general. I usually put most of my emotions into cooking, or doing something to let my anger out by breaking something or repeatedly hitting something, like boxing bag or copper box, breaking old glass bottles."
Learning and education environments	"It's kind of put me off balance when it came to school... I didn't really care... it put me off my education"<br><br>"lying in bed not doing anything"<br><br>"Half the things I should have learned, we didn't learn it."<br><br>"I started Uni in Covid… and that was pretty horrific… So in lockdown, and like halls. I was in like the eleventh floor of like a university

Lee J, Wong KK-Y. *BMJ Open* 2024; 14:e080161. doi: 10.1136/bmjopen-2023-080161

	building. So that was pretty rough, and it actually led to me dropping out of university.”
Mistrust	“I just didn’t like how they kept changing the rules, like one minute, there’s lockdown, then there’s not”<br><br>“stuff comes up, you start to question like “Oh, my gosh! Like, are you being? Are you telling the truth?” that, like “What can I believe? What is real like? What is just like? What is a control thing like? Is there a control thing like I don't know. Like should I be listening to these conspiracies? Is there some truth in them? Who am I supposed to listen to?”<br><br>“I am not left or right, I am in the middle and I don’t trust anyone.”<br><br>“I just don’t listen to it… you can recognising fearmongering, or tactics, whether that be through people or the government.”
Inequality and deprivation	“Cost of living? Can I mention that? The pandemic has caused inflation. For a young person, it is not easy anymore to do this, go to uni, apply for college. Even their parents are struggling, their parents can’t even afford for kids to go on a bus, pay for their travel, just to get an education and stuff like that, then you’re kind of going in a cycle.”<br><br>“I think financially, it was hard. Getting food, and you know, stuff like that. Um, so yeah… It also affects a lot of business like my dad's business. You know he has a shop and he; I remember, he was forced to shut down for a couple of days. And it kind of did affect him a lot.”

Lee J, Wong KK-Y. *BMJ Open* 2024; 14:e080161. doi: 10.1136/bmjopen-2023-080161

	"I had my nan next door and I just wanted to make sure she was alright at night, obviously with her depression and stuff, then I had my uncle in another room, and a lodger at the same time, there was a lot of us in the house, there wasn't as much as my mums there was still quite a few people, the house is quite small as well"
Loss of opportunities	"missing out on opportunities was probably the biggest thing that lockdown... did to me"<br><br>"I think Covid did like f*ck up a lot of my potential job opportunities"<br><br>"Covid definitely took away my ability to make – to make friends with people in organisations that I started around Covid"<br><br>"Before the pandemic, I used to be more active, and volunteer at my local college playground. Then since lockdown, I haven't volunteered. And I just haven't been as active, as going outside as I used to."
Grief and bereavement loss	"there's been quite a few deaths in our family"<br><br>"a lot of people lost close family or family friends... people are still grieving about it."<br><br>"a lot of people have lost their loved ones. That's affected them. Yeah, I feel like mental support would probably be the best in a pandemic."

Table D2. Sub-themes with supporting quotes on participant's support needs

Sub-theme	Illustrative quotes
Support for health	Mental health –<br>"at school… during mindfulness workshops… we've had a lot – it just didn't work"<br>"It can be like, literally, just being like heard and knowing that you're being heard."<br>"the problem with my school is, they never really had [mental health services]... I didn't have any options in school."<br>"groups need to be formed, so people can talk about it… I think that's a really good idea just to just even talk. Just let you just say whatever…"<br>"to build up their confidence and like, help them feel good about themselves"<br><br>Physical health -<br>"physical health, getting enough sleep, get enough exercise, getting enough water to drink, basically really just reminding us a lot of the time by sending us constant emails about how we should be taking care of ourselves."<br>"maybe, a regular check-up, to see how I am and how I was in the past. To see if there are any changes and to see what do from there, to see what I need to sort out."
Relational support	Friends -<br>"you go to friends... having... people or like a community that you feel safe and heard in."

	"my friends have like the biggest impact? … you can... feel like safe... they're not judging you"<br>"I got quite a lot of support from friends, because obviously they were quite worried about me not sleeping"<br><br>Family –<br>"my family... emotional support like talking on the phone a lot."<br>"my mum was very engaged with my home learning... I learned a lot... when we got back and we did the subject, I was at the top. So I knew why she did that."<br>"my family like support, you know, like emotional support like talking on the phone a lot."
Support for forming routines and developing new skills	"it's just my phone... but, you're creating habits. If I was to run one [workshop], it could be... teaching them what the algorithm is and what it does, and why... it's just getting them, a comprehensive understanding of like, this is what [social media] is. This is what it does. This is how you can prevent it."<br><br>"creativity-based workshops like, stuff like, even teaching people how to crochet."
Support for learning and education	"a lot of other people feel like actually there wasn't a lot of support like especially from our university."<br><br>"some teachers didn't really support us… I don't think there was much support from schools" (P115, 18 years, female)

	"I don't think I received any [support] if I am honest. The closest you could argue about support was 2 years after the pandemic for my year. I had Saturday school for extra support, but it was for only one subject."
Combating inequality and deprivation	"There is no one in that government who has come from the working-class… They think people go through what they have experienced as opposed to what is actually going on. Offering services and going into schools… when you're young, that needs to be followed by someone who knows. It's knowing that someone is there and that people can reach it. Some service needs to be formed that is implemented in unis, schools, colleges, so you know that someone is going to be there for you."<br><br>"obviously, the people that don't have technology that wanted to go on the team would get laptops from school… in Islington we did it really well. Like these kids still have the Chromebooks from the lockdown. My friends still have their Chromebook. So she still uses it for schoolwork now."

Appendix E: General developmental difficulties faced by young people during the Covid-19 pandemic

While not the focus of the current study, participants also discussed general developmental challenges unrelated to Covid-19. Four subordinate themes were identified under general difficulties:

general health difficulties (mental and physical health), general relational difficulties (friends and family), general difficulties at school, and careers and next steps.

Sub-theme	Illustrative quotes
General mental health difficulties	"I remember in Year 7 and Year 8, whenever I felt like, anxious about something… I don't even know what was going on – I don't know, growing up… something like that was just an experience"<br><br>"I feel like breaking down and like, my emotions (right) into tiny pieces (alright), more manageable pieces and I know if I keep breaking them down and keep whacking at it then eventually they'll become as if it's nothing."
General social difficulties	"I didn't have a lot of friends, and I still don't have a lot of friends... I am not that good at making new relationships."<br><br>"feel out of place – socially awkward or into a shopping mall that I've – I never go to and just feeling like it's scary because I don't fit in."
Careers and next steps	"the challenge of finding work... I was working the first half of 2020 during the pandemic, it kept me busy. But then I stopped, I was jobless for a year... When I got into work again, I wasn't interacting properly. I didn't know how to work."
General difficulties at school	"stuff that I find hardest like school work, it's like… just so hard. That's like, the biggest challenge actually."

	"I have put a lot of pressure on myself to get good grades because I have for a long time, so when I don't it's like, I feel like I've failed a lot."
General relationship with family	"my mum left when I was a little kid... she just stopped calling and every time she called I'd break down"
General physical health difficulties	"I've been on my period for like, a year straight, like, straight"<br><br>"I don't know what it is, but I have like pains like chest pains a lot, and I've been like to have my heart checks and everything"

Lee J, Wong KK-Y. *BMJ Open* 2024; 14:e080161. doi: 10.1136/bmjopen-2023-080161
